# 2,4-Bis(4-eth­oxy­phen­yl)-7-methyl-3-aza­bicyclo­[3.3.1]nonan-9-one

**DOI:** 10.1107/S1600536812006563

**Published:** 2012-02-17

**Authors:** Dong Ho Park, V. Ramkumar, P. Parthiban

**Affiliations:** a Department of Biomedicinal Chemistry, Inje University, Gimhae, Gyeongnam 621 749, Republic of Korea; b Department of Chemistry, IIT Madras, Chennai 600 036, TamilNadu, India

## Abstract

The mol­ecule of the title compound, C_25_H_31_NO_3_, exists in a twin-chair conformation with an equatorial orientation of the 4-eth­oxy­phenyl groups, as observed for its *ortho* isomer [Parthiban, Ramkumar, Park & Jeong (2011*b* ▶), *Acta Cryst.* E**67**, o1475–o1476]. The methyl and 4-eth­oxy­phenyl groups are also equatorially oriented on the bicycle, as in the *ortho* analogue. In particular, although the cyclo­hexa­none ring deviates from an ideal chair, the piperidone ring is closer to an ideal chair, whereas in the *ortho* isomer both rings are significantly puckered and deviate from ideal chairs. The 4-eth­oxy­phenyl groups on both sides of the secondary amine group are oriented at an angle of 26.11 (3)° with respect to each other, but the 2-eth­oxy­phenyl groups in the *ortho* isomer are oriented by less than half this [12.41 (4)°]. In contrast to the absence of any significant inter­actions in the crystal packing of the *ortho* isomer, the title compound features N—H⋯O inter­actions, linking the mol­ecules along the *b* axis.

## Related literature

For the synthesis and stereochemistry of 3-aza­bicyclo­[3.3.1] nonan-9-ones, see: Park *et al.* (2011 ▶). For the biological activities of 3-aza­bicyclo­[3.3.1]nonan-9-ones, see: Barker *et al.* (2005 ▶); Parthiban *et al.* (2009 ▶, 2010*a* ▶,*b* ▶,2011*a* ▶). For a related structure, see: Parthiban *et al.* (2011*b* ▶). For ring-puckering parameters, see: Cremer & Pople (1975 ▶); Nardelli (1983 ▶).
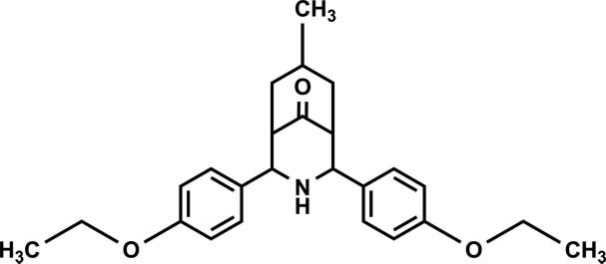


## Experimental

### 

#### Crystal data

C_25_H_31_NO_3_
*M* *_r_* = 393.51
Orthorhombic, 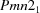

*a* = 19.329 (4) Å
*b* = 6.7967 (12) Å
*c* = 8.2501 (16) Å
*V* = 1083.8 (4) Å^3^
*Z* = 2
Mo *K*α radiation
μ = 0.08 mm^−1^
*T* = 298 K
0.35 × 0.28 × 0.10 mm

#### Data collection

Bruker APEXII CCD area-detector diffractometer
Absorption correction: multi-scan (*SADABS*; Bruker, 2004 ▶) *T*_min_ = 0.973, *T*_max_ = 0.992
1565 measured reflections
1165 independent reflections
950 reflections with *I* > 2σ(*I*)
*R*_int_ = 0.015

#### Refinement

*R*[*F* ^2^ > 2σ(*F* ^2^)] = 0.037
*wR*(*F* ^2^) = 0.094
*S* = 1.05
1165 reflections
150 parameters
4 restraints
H atoms treated by a mixture of independent and constrained refinement
Δρ_max_ = 0.12 e Å^−3^
Δρ_min_ = −0.16 e Å^−3^

### 

Data collection: *APEX2* (Bruker, 2004 ▶); cell refinement: *APEX2* and *SAINT-Plus* (Bruker, 2004 ▶); data reduction: *SAINT-Plus* and *XPREP* (Bruker, 2004 ▶); program(s) used to solve structure: *SHELXS97* (Sheldrick, 2008 ▶); program(s) used to refine structure: *SHELXL97* (Sheldrick, 2008 ▶); molecular graphics: *ORTEP-3* (Farrugia, 1997 ▶); software used to prepare material for publication: *SHELXL97*.

## Supplementary Material

Crystal structure: contains datablock(s) global, I. DOI: 10.1107/S1600536812006563/bq2338sup1.cif

Structure factors: contains datablock(s) I. DOI: 10.1107/S1600536812006563/bq2338Isup2.hkl

Supplementary material file. DOI: 10.1107/S1600536812006563/bq2338Isup3.cml

Additional supplementary materials:  crystallographic information; 3D view; checkCIF report

## Figures and Tables

**Table 1:** Hydrogen-bond geometry (Å, °)

*D*—H⋯*A*	*D*—H	H⋯*A*	*D*⋯*A*	*D*—H⋯*A*
N1—H1*A*⋯O1^i^	0.86 (2)	2.26 (2)	3.073 (4)	158 (3)

Symmetry code: (i) 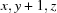.

**Fig. 1.**
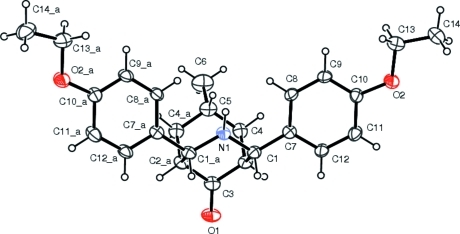
Molecular structure of the title compound showing the atom labeling scheme. Displacement ellipsoids are drawn at the 30% probability level. Symmetry code for the half part of the molecule: -x, y, z.

**Fig. 2.**
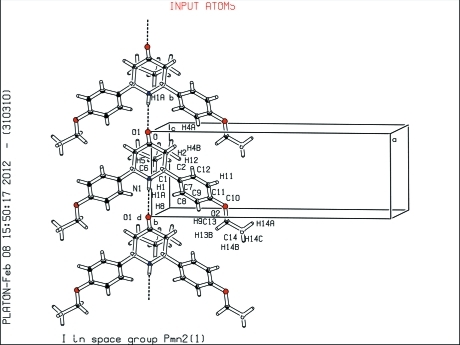
The packing of the title compound along b-axis. Dashed line shows intermolecular N—H···O H-bonds.

## supplementary crystallographic information

### Comment

The 3-azabicyclononane nucleus is an important class of pharmacophore due to its broad-spectrum of biological actions ranging from antibacterial to anticancer (Barker *et al.*, 2005; Parthiban *et al.*, 2009, 2010*a*,*b*, 2011a). Owing to their broad-spectrum of biological actions, synthesis as well as isolaton of new molecules from the natural products, and their stereochemical analysis are considered as important in the field of medicinal chemistry. Hence, we synthesized the title compound by a modified and an optimized successive double Mannich condensation. Thus the obtained crystal was undertaken for this study to explore its stereochemistry in the solid-state.

The crystallographic parameters *viz.* torsion angles, asymmetry parameters and ring puckering parameters calculated for the title compound show that the piperidone ring adopts a near ideal chair conformation, according to Cremer & Pople and Nardelli (Fig. 1). The total puckering amplitude, Q_T_ is 0.607 (6) Å, the phase angle θ is 7.7 (6)° and φ is 180.0° (Cremer & Pople, 1975). The smallest displacement asymmetry parameters q_2_ and q_3_ are 0.081 (6) Å and 0.601 (6) Å, respectively (Nardelli, 1983). On the other hand, the cyclohexane ring deviates from the ideal chair conformation by Q_T_ = 0.536 (6), θ = 170.2 (7)° and φ = 240.0° (Cremer & Pople, 1975) as well as Nardelli by q_2_ = 0.092 (7) and q_3_ = 0.528 (6)° (Nardelli, 1983). In its *ortho* isomer, that is, 2,4-*bis*(2-ethoxyphenyl)-7-methyl-3-azabicyclo[3.3.1]nonan-9-one (Parthiban *et al.*, 2011b), both the piperidone and cyclohexanone rings deviated the ideal chair as follows (Q_T_ = 0.5889 (18), θ = 7.19 (18)° and Q_T_ = 0.554 (2), θ = 12.2 (2)°, respectively).

The aryl groups are orientated at an angle of 26.11 (3)° to each other. The center of symmetry passes through C6 C5 C3 N1 and O1. The torsion angle of C3—C2—C1—C7 and its mirror image is -176.7 (5)°. The angle with C&P plane normal of bonds C1—C7 as well as C1a—C7a and C5—C6 are 73.27 and 65.36 (2), respectively, conforms the equatorial disposition of the aryl and alkyl groups on the bicycle. Hence, the title compound C_25_H_31_NO_3_, exists in a double-chair conformation with an equatorial orientation of the 4-ethoxyphenyl groups on both sides of the secondary amino group on the heterocycle and exocyclic orientation of the methyl on the cyclohexane ring.

The crystal packing is stabilized by an intermolecular N—H···O interaction of 2.26 (2) Å (Table 1 and Fig. 2).

### Experimental

The 2,4-*bis*(4-ethoxyphenyl)-7-methyl-3-azabicyclo[3.3.1]nonan-9-one was synthesized by a modified and an optimized Mannich condensation in one-pot, using 4-ethoxybenzaldehyde (0.1 mol, 15.018 g/13.91 ml), cyclohexanone (0.05 mol, 5.61 g/6.14 ml) and ammonium acetate (0.075 mol, 5.78 g) in a 50 ml of absolute ethanol (Park *et al.*, 2001). The mixture was gently warmed on a hot plate at 303–308 K (30–35° C) with moderate stirring till the complete consumption of the starting materials, which was monitored by TLC. At the end, the crude azabicyclic ketone was separated by filtration and gently washed with 1:5 cold ethanol–ether mixture. X-ray diffraction quality crystals of the title compound were obtained by slow evaporation from ethanol.

### Refinement

The nitrogen H atom and C6 H atoms were located by difference Fourier map and refined isotropically. Other H atoms were fixed geometrically and allowed to ride on the parent C atoms with aromatic C—H = 0.93 Å, aliphatic C—H = 0.98 Å and methylene C—H = 0.97 Å. The displacement parameters were set for phenyl, methylene and aliphatic H atoms at *U*_iso_(H) = 1.2*U*_eq_(C) and for methyl H atoms at *U*_iso_(H) = 1.5*U*_eq_(C). Because of the meaningless of the absolute structure parameter, 400 Friedel-pairs were merged before final refinement

### Crystal data

**Table:**

C_25_H_31_NO_3_	*F*(000) = 424
*M**_r_* = 393.51	*D*_x_ = 1.206 Mg m^−^^3^
Orthorhombic, *P**m**n*2_1_	Mo *K*α radiation, λ = 0.71073 Å
Hall symbol: P 2ac -2	Cell parameters from 1281 reflections
*a* = 19.329 (4) Å	θ = 2.5–22.3°
*b* = 6.7967 (12) Å	µ = 0.08 mm^−^^1^
*c* = 8.2501 (16) Å	*T* = 298 K
*V* = 1083.8 (4) Å^3^	Block, colourless
*Z* = 2	0.35 × 0.28 × 0.10 mm

### Data collection

**Table:**

Bruker APEXII CCD area-detector diffractometer	1165 independent reflections
Radiation source: fine-focus sealed tube	950 reflections with *I* > 2σ(*I*)
Graphite monochromator	*R*_int_ = 0.015
φ and ω scans	θ_max_ = 26.0°, θ_min_ = 2.1°
Absorption correction: multi-scan (*SADABS*; Bruker, 2004)	*h* = 0→23
*T*_min_ = 0.973, *T*_max_ = 0.992	*k* = 0→8
1565 measured reflections	*l* = −7→10

### Refinement

**Table:**

Refinement on *F*^2^	Primary atom site location: structure-invariant direct methods
Least-squares matrix: full	Secondary atom site location: difference Fourier map
*R*[*F*^2^ > 2σ(*F*^2^)] = 0.037	Hydrogen site location: inferred from neighbouring sites
*wR*(*F*^2^) = 0.094	H atoms treated by a mixture of independent and constrained refinement
*S* = 1.05	*w* = 1/[σ^2^(*F*_o_^2^) + (0.0502*P*)^2^ + 0.0596*P*] where *P* = (*F*_o_^2^ + 2*F*_c_^2^)/3
1165 reflections	(Δ/σ)_max_ < 0.001
150 parameters	Δρ_max_ = 0.12 e Å^−^^3^
4 restraints	Δρ_min_ = −0.16 e Å^−^^3^

### Special details

**Table:**

Geometry. All esds (except the esd in the dihedral angle between two l.s. planes) are estimated using the full covariance matrix. The cell esds are taken into account individually in the estimation of esds in distances, angles and torsion angles; correlations between esds in cell parameters are only used when they are defined by crystal symmetry. An approximate (isotropic) treatment of cell esds is used for estimating esds involving l.s. planes.
Refinement. Refinement of *F*^2^ against ALL reflections. The weighted *R*-factor *wR* and goodness of fit *S* are based on *F*^2^, conventional *R*-factors *R* are based on *F*, with *F* set to zero for negative *F*^2^. The threshold expression of *F*^2^ > σ(*F*^2^) is used only for calculating *R*-factors(gt) *etc*. and is not relevant to the choice of reflections for refinement. *R*-factors based on *F*^2^ are statistically about twice as large as those based on *F*, and *R*-factors based on ALL data will be even larger.

### Fractional atomic coordinates and isotropic or equivalent isotropic displacement parameters (Å^2^)

**Table:**

	*x*	*y*	*z*	*U*_iso_*/*U*_eq_	
C1	0.06340 (12)	0.4381 (3)	0.8529 (3)	0.0363 (6)	
H1	0.0632	0.4085	0.7366	0.044*	
C2	0.06391 (13)	0.2389 (3)	0.9456 (3)	0.0389 (6)	
H2	0.1044	0.1628	0.9112	0.047*	
C3	0.0000	0.1332 (5)	0.8932 (5)	0.0415 (8)	
C4	0.06491 (14)	0.2575 (4)	1.1304 (3)	0.0477 (7)	
H4A	0.0714	0.1276	1.1767	0.057*	
H4B	0.1044	0.3370	1.1615	0.057*	
C5	0.0000	0.3480 (6)	1.2034 (4)	0.0489 (10)	
H5	0.0000	0.4887	1.1772	0.059*	
C6	0.0000	0.3276 (10)	1.3882 (6)	0.0786 (15)	
C7	0.12754 (11)	0.5559 (3)	0.8882 (3)	0.0349 (6)	
C8	0.12963 (12)	0.7124 (4)	0.9938 (3)	0.0397 (6)	
H8	0.0895	0.7477	1.0489	0.048*	
C9	0.18980 (12)	0.8191 (4)	1.0205 (3)	0.0434 (6)	
H9	0.1898	0.9248	1.0921	0.052*	
C10	0.24949 (12)	0.7671 (4)	0.9402 (3)	0.0415 (6)	
C11	0.24911 (14)	0.6067 (4)	0.8392 (4)	0.0517 (8)	
H11	0.2898	0.5676	0.7885	0.062*	
C12	0.18887 (13)	0.5027 (4)	0.8122 (4)	0.0463 (6)	
H12	0.1893	0.3956	0.7421	0.056*	
C13	0.31170 (14)	1.0533 (4)	1.0305 (4)	0.0610 (8)	
H13A	0.3057	1.0357	1.1463	0.073*	
H13B	0.2741	1.1343	0.9905	0.073*	
C14	0.37939 (15)	1.1511 (5)	0.9972 (5)	0.0731 (10)	
H14A	0.4162	1.0730	1.0414	0.110*	
H14B	0.3798	1.2790	1.0466	0.110*	
H14C	0.3856	1.1643	0.8823	0.110*	
N1	0.0000	0.5455 (4)	0.8897 (4)	0.0361 (7)	
O1	0.0000	−0.0133 (4)	0.8084 (4)	0.0603 (8)	
O2	0.31106 (9)	0.8665 (3)	0.9511 (3)	0.0599 (6)	
H1A	0.0000	0.656 (3)	0.840 (4)	0.033 (10)*	
H6A	−0.0436 (13)	0.385 (5)	1.427 (5)	0.091 (12)*	
H6B	0.0000	0.189 (4)	1.421 (7)	0.089 (18)*	

### Atomic displacement parameters (Å^2^)

**Table:**

	*U*^11^	*U*^22^	*U*^33^	*U*^12^	*U*^13^	*U*^23^
C1	0.0373 (14)	0.0399 (13)	0.0318 (12)	0.0017 (10)	0.0031 (10)	0.0015 (11)
C2	0.0344 (13)	0.0350 (12)	0.0471 (13)	0.0070 (10)	0.0024 (12)	0.0050 (13)
C3	0.050 (2)	0.0315 (18)	0.0426 (18)	0.000	0.000	0.0048 (19)
C4	0.0452 (17)	0.0520 (15)	0.0459 (14)	−0.0029 (12)	−0.0086 (12)	0.0147 (14)
C5	0.057 (3)	0.053 (2)	0.0369 (18)	0.000	0.000	0.005 (2)
C6	0.095 (4)	0.103 (4)	0.038 (2)	0.000	0.000	0.008 (3)
C7	0.0319 (13)	0.0376 (13)	0.0353 (12)	0.0030 (10)	0.0060 (10)	0.0055 (12)
C8	0.0310 (12)	0.0453 (13)	0.0428 (13)	0.0050 (10)	0.0055 (10)	−0.0038 (13)
C9	0.0406 (14)	0.0435 (13)	0.0461 (13)	0.0031 (11)	0.0023 (12)	−0.0054 (14)
C10	0.0334 (13)	0.0414 (13)	0.0498 (13)	−0.0003 (11)	0.0052 (12)	−0.0004 (13)
C11	0.0369 (15)	0.0466 (15)	0.0717 (19)	0.0010 (12)	0.0197 (14)	−0.0083 (16)
C12	0.0442 (16)	0.0390 (13)	0.0556 (14)	0.0017 (12)	0.0131 (12)	−0.0078 (14)
C13	0.0543 (18)	0.0615 (18)	0.0672 (19)	−0.0074 (13)	0.0053 (15)	−0.0171 (18)
C14	0.065 (2)	0.076 (2)	0.079 (2)	−0.0226 (17)	0.0056 (18)	−0.021 (2)
N1	0.0317 (16)	0.0324 (16)	0.0441 (16)	0.000	0.000	0.0075 (14)
O1	0.073 (2)	0.0366 (14)	0.0713 (18)	0.000	0.000	−0.0071 (16)
O2	0.0388 (10)	0.0547 (11)	0.0862 (14)	−0.0084 (9)	0.0101 (10)	−0.0158 (13)

### Geometric parameters (Å, º)

**Table:**

C1—N1	1.458 (3)	C8—C9	1.388 (3)
C1—C7	1.504 (3)	C8—H8	0.9300
C1—C2	1.555 (3)	C9—C10	1.376 (3)
C1—H1	0.9800	C9—H9	0.9300
C2—C3	1.493 (3)	C10—O2	1.371 (3)
C2—C4	1.530 (4)	C10—C11	1.373 (4)
C2—H2	0.9800	C11—C12	1.380 (3)
C3—O1	1.216 (4)	C11—H11	0.9300
C3—C2^i^	1.493 (3)	C12—H12	0.9300
C4—C5	1.521 (4)	C13—O2	1.429 (3)
C4—H4A	0.9700	C13—C14	1.493 (4)
C4—H4B	0.9700	C13—H13A	0.9700
C5—C4^i^	1.521 (4)	C13—H13B	0.9700
C5—C6	1.531 (6)	C14—H14A	0.9600
C5—H5	0.9800	C14—H14B	0.9600
C6—H6A	0.980 (18)	C14—H14C	0.9600
C6—H6B	0.98 (2)	N1—C1^i^	1.458 (3)
C7—C8	1.375 (3)	N1—H1A	0.855 (18)
C7—C12	1.389 (3)		
			
N1—C1—C7	112.68 (19)	C12—C7—C1	118.5 (2)
N1—C1—C2	109.8 (2)	C7—C8—C9	122.0 (2)
C7—C1—C2	111.28 (19)	C7—C8—H8	119.0
N1—C1—H1	107.6	C9—C8—H8	119.0
C7—C1—H1	107.6	C10—C9—C8	119.4 (2)
C2—C1—H1	107.6	C10—C9—H9	120.3
C3—C2—C4	109.8 (2)	C8—C9—H9	120.3
C3—C2—C1	105.8 (2)	O2—C10—C11	115.8 (2)
C4—C2—C1	114.7 (2)	O2—C10—C9	124.7 (2)
C3—C2—H2	108.8	C11—C10—C9	119.5 (2)
C4—C2—H2	108.8	C10—C11—C12	120.6 (2)
C1—C2—H2	108.8	C10—C11—H11	119.7
O1—C3—C2	124.08 (16)	C12—C11—H11	119.7
O1—C3—C2^i^	124.08 (16)	C11—C12—C7	120.9 (3)
C2—C3—C2^i^	111.6 (3)	C11—C12—H12	119.5
C5—C4—C2	114.6 (2)	C7—C12—H12	119.5
C5—C4—H4A	108.6	O2—C13—C14	108.6 (2)
C2—C4—H4A	108.6	O2—C13—H13A	110.0
C5—C4—H4B	108.6	C14—C13—H13A	110.0
C2—C4—H4B	108.6	O2—C13—H13B	110.0
H4A—C4—H4B	107.6	C14—C13—H13B	110.0
C4^i^—C5—C4	111.1 (3)	H13A—C13—H13B	108.3
C4^i^—C5—C6	110.9 (2)	C13—C14—H14A	109.5
C4—C5—C6	110.9 (2)	C13—C14—H14B	109.5
C4^i^—C5—H5	107.9	H14A—C14—H14B	109.5
C4—C5—H5	107.9	C13—C14—H14C	109.5
C6—C5—H5	107.9	H14A—C14—H14C	109.5
C5—C6—H6A	107 (3)	H14B—C14—H14C	109.5
C5—C6—H6B	111 (4)	C1^i^—N1—C1	114.3 (3)
H6A—C6—H6B	107 (3)	C1^i^—N1—H1A	109.9 (10)
C8—C7—C12	117.5 (2)	C1—N1—H1A	109.9 (10)
C8—C7—C1	124.0 (2)	C10—O2—C13	118.37 (19)
			
N1—C1—C2—C3	57.5 (3)	C12—C7—C8—C9	2.2 (4)
C7—C1—C2—C3	−177.0 (2)	C1—C7—C8—C9	−178.9 (2)
N1—C1—C2—C4	−63.6 (3)	C7—C8—C9—C10	−0.4 (4)
C7—C1—C2—C4	61.9 (3)	C8—C9—C10—O2	176.9 (2)
C4—C2—C3—O1	−125.3 (4)	C8—C9—C10—C11	−2.1 (4)
C1—C2—C3—O1	110.4 (4)	O2—C10—C11—C12	−176.3 (3)
C4—C2—C3—C2^i^	59.6 (4)	C9—C10—C11—C12	2.8 (4)
C1—C2—C3—C2^i^	−64.7 (3)	C10—C11—C12—C7	−1.0 (4)
C3—C2—C4—C5	−53.1 (3)	C8—C7—C12—C11	−1.5 (4)
C1—C2—C4—C5	65.9 (3)	C1—C7—C12—C11	179.5 (2)
C2—C4—C5—C4^i^	46.1 (4)	C7—C1—N1—C1^i^	178.43 (16)
C2—C4—C5—C6	170.0 (3)	C2—C1—N1—C1^i^	−56.9 (3)
N1—C1—C7—C8	22.0 (3)	C11—C10—O2—C13	169.0 (3)
C2—C1—C7—C8	−101.9 (3)	C9—C10—O2—C13	−10.0 (4)
N1—C1—C7—C12	−159.1 (2)	C14—C13—O2—C10	−168.2 (3)
C2—C1—C7—C12	77.1 (3)		

Symmetry code: (i) −*x*, *y*, *z*.

### Hydrogen-bond geometry (Å, º)

**Table:**

*D*—H···*A*	*D*—H	H···*A*	*D*···*A*	*D*—H···*A*
N1—H1*A*···O1^ii^	0.86 (2)	2.26 (2)	3.073 (4)	158 (3)

Symmetry code: (ii) *x*, *y*+1, *z*.
